# Outcome of offenders with substance use disorder

**DOI:** 10.1192/j.eurpsy.2023.1402

**Published:** 2023-07-19

**Authors:** R. Felhi, G. Hamdi, S. Chebli, R. Ridha

**Affiliations:** 1 Razi hospital, Manouba

## Abstract

**Introduction:**

Substance use is related to higher risk of criminality among prisoner with or without co-occurring mental disorder. This contributes to a poor correctional outcome and higher risk of suicide and other psychiatric disorders.

**Objectives:**

The aim of this study is to compare offenders with substance use disorder with offenders with no addictive comorbidity.

**Methods:**

We studied the medical files of all the offenders referred to the forensic psychiatry unit in the Razi hospital for an examination between January 2010 and October 2020 analyzed sociodemographic, psychiatric and criminological factors of this population.

**Results:**

We found that the first group, with substance use disorder, was substantially consisted of male offenders (99.2%) compared to the second group (92%); (p=0). A significantly higher level of education was found in the second group compared to the first (p=0.004). Offenders with no substance use disorder tended to live with their family while the rest of the cases lived on their own (p=0.018), they also had three times more family history of psychiatric disorders than the other group (p=0). Psychiatric comorbidity was more important in the second group with more cases of schizophrenia and mental disability while the first group consisted mainly of people with personality disorders (p=0.018).

The average number of anterior convictions was 3.12 first group and 1.33 in the second group, which is significantly lower (p=0)

**Conclusions:**

Specific interventions, in particular addiction management, are required to reduce the criminal risk in this population.

**Disclosure of Interest:**

None Declared

